# Synthesis of morphologically diverse dual thermo- and pH-responsive nano-objects *via* RAFT-mediated emulsion PISA

**DOI:** 10.1039/d5na00779h

**Published:** 2025-10-10

**Authors:** Svetlana Lukáš Petrova, Ewa Pavlova, Martin Hrubý, Václav Pokorný

**Affiliations:** a Institute of Macromolecular Chemistry v.v.i., Academy of Sciences of the Czech Republic Heyrovsky Sq. 2 162 06 Prague 6 Czech Republic petrova@imc.cas.cz

## Abstract

We report a versatile and efficient strategy for the synthesis of dual thermo- and pH-responsive polymeric nano-objects with rich morphological diversity, achieved *via* RAFT-mediated aqueous emulsion polymerization-induced self-assembly (PISA). Using a thermoresponsive poly(triethylene glycol methyl ether methacrylate) (PTEGMA) macro-chain transfer agent and a pH-sensitive diisopropylaminoethyl methacrylate (DPA) monomer, we generated PTEGMA-*b*-PDPA diblock copolymer nano-objects under conditions both below and above the LCST of PTEGMA. By systematically varying the length of the PDPA block, we accessed a wide array of morphologies—including micelles, worms, vesicles, and intricate “octopus”-like structures—simply by adjusting the polymerization temperature and hydrophobic block length. These nanostructures displayed clear dual responsiveness: thermally triggered aggregation around ∼40 °C and reversible disassembly in acidic environments. Our findings highlight the precise morphological control achievable through aqueous RAFT-PISA and underscore its potential for designing smart nanomaterials tailored for biomedical and stimuli-responsive applications.

## Abstract 2

Efficient synthesis of dual thermo- and pH-responsive polymeric nano-objects *via* RAFT-mediated PISA using PTEGMA and PDPA, yielding tunable micelles, worms, “octopus”-like structures, and vesicles reversible to heat (∼40 °C) and acidic conditions.

## Graphical abstract

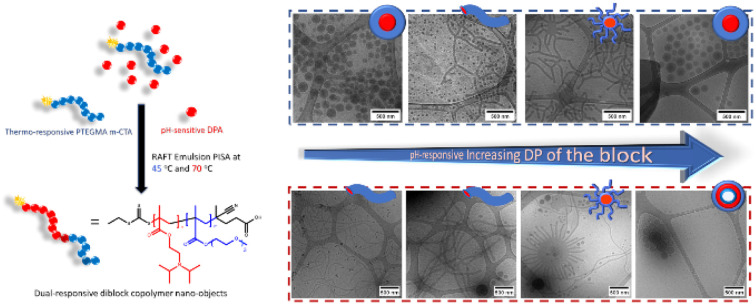

## 1. Introduction

In the past few years, polymerization-induced self-assembly (PISA) has become a widely recognized and efficient method for the deliberate creation of block copolymer nanostructures.^1–5^ Fundamentally, PISA involves the chain growth of a soluble precursor polymer block by adding an insoluble second block in a selective solvent environment, commonly water. As this process occurs, the resulting amphiphilic diblock copolymers (AmCPs) spontaneously organize into various nanostructured morphologies, including micelles, worms, and/or vesicles in a well-controlled manner.^6–8^ PISA is compatible with various “living” or controlled polymerization methods,^9–11^ carried out by either dispersion or emulsion polymerization in the reaction medium. Both techniques enable the efficient production of various nanoparticles (NPs) at relatively high solid concentrations (up to 50 wt%), often eliminating the need for additional purification steps.^12–15^ Indeed, reversible addition–fragmentation chain transfer (RAFT) dispersion polymerization (RAFTDP) is particularly effective in producing nano-objects with diverse and intricate morphologies—such as micelles, worms, and vesicles—that exhibit uniform physicochemical properties.^5,10,16,17^ In contrast, aqueous emulsion polymerization involves extending a water-soluble polymer chain with a monomer that is immiscible with water.^18–20^ This method provides benefits such as high polymerization rates and high monomer conversion, even at very high monomer concentrations.^21–24^

Stimuli-responsive nano-objects are well known from post-polymerization methods like nanoprecipitation, but remain rarely reported for PISA-derived NPs over the past decade, and there has been significant interest in stimuli-responsive polymers or “smart materials” with multiple functional groups, capable of adapting to environmental changes, due to their wide array of applications.^25^ In particular, the incorporation of stimuli-responsive functional groups into polymers has garnered significant interest within the context of PISA,^26,27^ as a means to precisely modulate the nanoparticle shape and size. Various external stimuli have been employed to trigger these transformations, including light,^27–29^ temperature,^30–32^ and pH.^24,33^ There has been growing interest in dual thermo- and pH-responsive materials for biomedical applications.^34–38^ However, only a few studies have investigated NPs that respond to both stimuli through PISA-based synthesis.^36,39–41^ Lovett *et al.* studied the pH- and thermo-responsive behavior of non-ionic vesicles made from glycerol monomethacrylate (GMA) and 2-hydroxypropyl methacrylate (HPMA). Using a carboxylic acid-functionalized RAFT agent, pH sensitivity arose from ionization of a single terminal carboxyl group on each PGMA stabilizer block, demonstrating the subtle worm-to-sphere transition. Notably, applying either a pH or temperature change induced a vesicle-to-sphere transformation.^42^ More recently, Rieger J. *et al.*^4^ reported a straightforward RAFT-mediated PISA in water that produced dual thermo- and pH-responsive nano-objects by copolymerizing *N*-cyanomethylacrylamide (PCMAm) with acrylic acid (AA). Particle morphology was determined by the core block length and AA content, with post-polymerization increases in AA ionization driving morphological transitions between vesicles, worms, and spheres. Temperature-dependent scattering measurements revealed complex thermoresponsive behavior, characterized by distinct cloud and clearing points corresponding to aggregation and polymer chain dissolution.

**Scheme 1:**
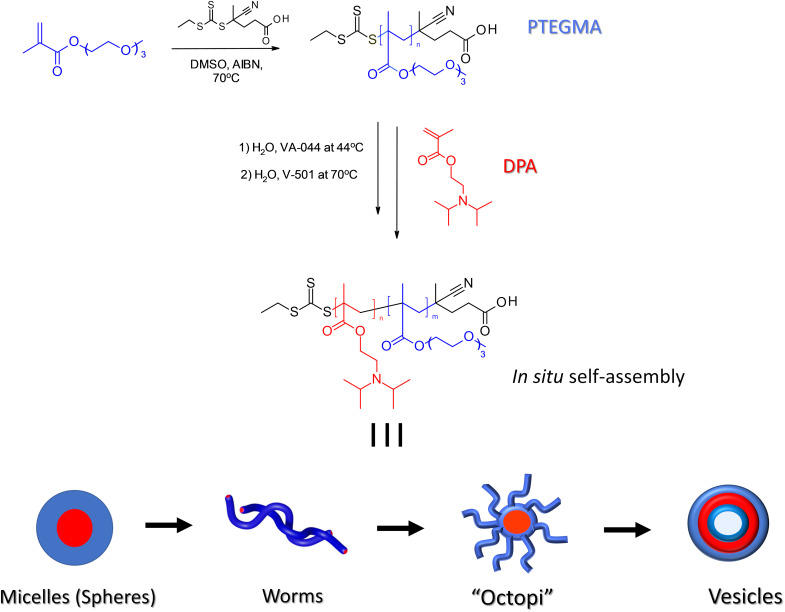
Schematic overview of the aqueous emulsion RAFT/PISA process for fabricating dual thermo- and pH-responsive PTEGMA-*b*-PDPA diblock copolymer nanoassemblies at polymerization temperatures of 45 °C and 70 °C.

Herein, we present a straightforward and effective strategy for the fabrication of nano-objects exhibiting sophisticated and tunable dual responsiveness to two distinct external stimuli: temperature and pH. To the best of our knowledge, this study is the first to combine thermoresponsive poly(triethylene glycol methyl ether methacrylate) (PTEGMA) and pH-responsive poly[2-(diisopropylamino)ethyl methacrylate] (PDPA) for the *in situ* synthesis of dual thermo-/pH-responsive PTEGMA-*b*-PDPA NPs, as illustrated in Scheme 1. This was successfully achieved *via* RAFT-mediated aqueous emulsion PISA at 10 wt% solids, conducted at temperatures both below and above the LCST-type cloud point (*T*_cp_) of PTEGMA in aqueous media (pH ∼ 8–9). This approach allowed systematic variation of the hydrophobic, pH-responsive PDPA block degree of polymerization (DP_*n*_ = 35, 85, 170, and 265). These assemblies exhibit properties highly advantageous for biomedical applications. In particular, PTEGMA offers a tunable *T*_cp_ around 50 °C, adjustable through copolymerization with hydrophobic comonomers.^43^ Meanwhile, the tertiary amine group in PDPA enables modulation of positive charge *via* protonation in response to pH changes. Specifically, lowering the pH below PDPA's p*K*_a_ (∼6.2–6.3) with HCl renders the DPA units hydrophilic a feature that enhances the pH sensitivity of PDPA-based NPs and supports targeted drug delivery to tumor sites through controlled drug encapsulation.^44,45^

A crucial aspect of this research is the demonstrated influence of polymerization temperature during PISA and the PDPA block length on the colloidal stability and morphology of the resulting NPs. At polymerization temperatures below the *T*_cp_ (∼45 °C), well-defined spherical structures predominantly formed, with the exception of PTEGMA_78_-*b*-PDPA_85_ and PTEGMA_78_-*b*-PDPA_170_, which self-assembled into worm-like and more complex “octopus-like” morphologies. Conversely, polymerization conducted above the *T*_cp_ (∼70 °C) produced a diverse range of nanoformulations, including worm-like micelles, highly branched intermediates as “octopus”, and vesicles. This exquisite morphological tunability underscores the strong synergistic interplay between thermo- and pH-responsive blocks within a single-step PISA process. Comprehensive characterization of the nano-objects was performed using cryogenic transmission electron microscopy (cryo-TEM), dynamic light scattering (DLS), and small-angle X-ray scattering (SAXS), providing detailed insights into their structural evolution and responsive behaviors.

## 2. Experimental section

### 2.1. Materials

Triethylene glycol methyl ether methacrylate (TEGMA, 93%, stabilized with MEHQ) was purified prior to use by stirring with an inhibitor remover (Aldrich #311332) for five minutes, followed by filtration to remove the inhibitor. 4-Cyano-4-(((ethylthio)carbonothioyl)thio)pentanoic acid (CETPA) was synthesized as previously reported^46^ (Scheme S1 and Fig. S1, in the SI). The initiators 2,2′-azobis(2-methylpropionitrile) (AIBN, 98%), 2,2′-azobis[2-(2-imidazolin-2yl)propane]dihydrochloride (VA-044, ≥98%) and 4,4′-azobis(4-cyanovaleric acid) (V-501, ≥98%) were used as received. 2-(Diisopropylamino)ethyl methacrylate (DPA, 97%) and dimethyl sulfoxide (DMSO, anhydrous, ≥99.8%) were distilled under an argon atmosphere prior to use. All chemicals were purchased from Sigma-Aldrich Ltd (Czech Republic).

### 2.2. Characterization techniques

A comprehensive explanation of the employed characterization techniques is provided in detail within the SI.

### 2.3. Synthesis of PTEGMA_78_-mCETPA (Scheme 1)

**Fig. 1:**
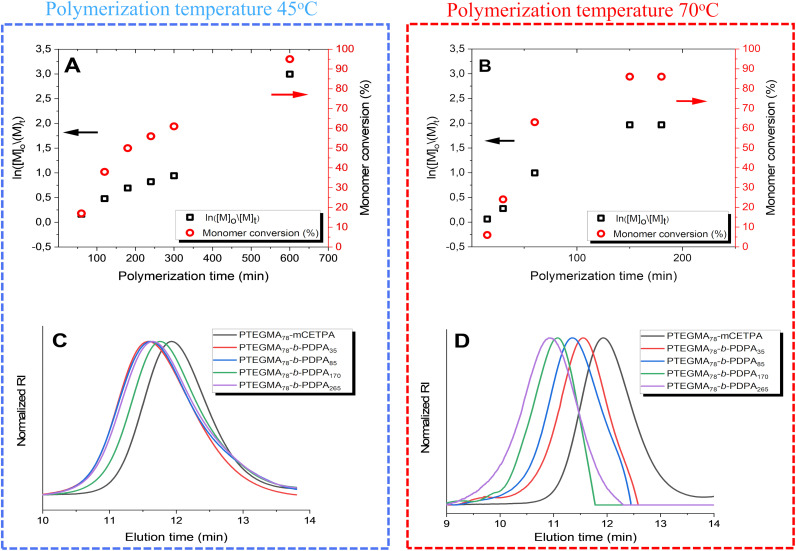
Monomer conversion *vs.* time profiles (red circles) alongside semi-logarithmic plots (black squares) for PTEGMA_78_-*b*-PDPA_265_ NPs polymerized at 45 °C (A) and 70 °C (B). Normalized SEC traces of the PTEGMA_78_-mCETPA (black curve) and the corresponding PTEGMA_78_-*b*-PDPA_*n*_ NPs obtained at 45 °C (C) and 70 °C (D), respectively. All SEC analyses were performed in DMF containing LiBr (1 g L^−1^) (see Table 1).

A typical protocol for the synthesis of PTEGMA_78_-mCETPA is outlined below: TEGMA (1.00 g, 4.31 mmol), CETPA (18.27 mg for DP_*n*_ 78), and AIBN (2.28 mg) were accurately weighed and dissolved in anhydrous DMSO (6.0 mL) in a 20 mL round-bottom flask. The reaction mixture was purged with Ar for 30 minutes. Subsequently, the flask was immersed in a preheated oil bath set at 70 °C. To determine the monomer conversion, aliquots were periodically taken from the reaction medium and analyzed by ^1^H NMR. Then the polymerization was quenched by cooling the flask in an ice-water bath and exposing the mixture to air. The crude homopolymer was precipitated by adding the reaction mixture dropwise into cold *n*-hexane (100 mL), followed by centrifugation to isolate the solid. Then it was redissolved in a minimal amount of 1,4-dioxane and purified by Sephadex LH-20 column chromatography using 1,4-dioxane as the eluent. The solvent was removed by rotary evaporation under reduced pressure, and the purified product was vacuum-dried to yield a yellow, viscous material. The resulting PTEGMA_78_-mCETPA was characterized by ^1^H NMR spectroscopy, (Fig. S2, in the SI). Size exclusion chromatography (SEC) was performed in DMF containing LiBr (1 g L^−1^) and calibrated against poly(methyl methacrylate) (PMMA) standards, as shown by the black curve in panels A and B of Fig. 1. The theoretical number-average molecular weight (*M*_n_,_calc_) calculated using eqn (1) is 18 100. The SEC results indicate an *M*_n_,SEC of 18 000 with a dispersity (*Đ*) of 1.13; the theoretical number-average molecular weight (*M*_n_,_calc_) was calculated using eqn (1):.

1 *M*_n,calc_ = [TEGMA]_0_/[RAFT]_0_ × *M*_TEGMA_ + *M*_CETPA_

where *M*_TEGMA_ and *M*_CETPA_ represent the molecular weights of the TEGMA and chain transfer agent (CETPA), respectively. [TEGMA]_0_ and [RAFT]_0_ denote the initial concentrations of the TEGMA and CETPA, respectively.

### 2.4. PISA synthesis of PTEGMA_78_-*b*-PDPA_*n*_ NPs at temperatures below and above the *T*_cp_ of PTEGMA_78_-mCETPA in water (Scheme 1)

PISA RAFT polymerization at a solid concentration of 10 wt% in aqueous solution was carried out as follows: in a 5 or 10 mL vial equipped with a magnetic stirrer, PTEGMA_78_-mCETPA (80 mg, 0.005 mmol) was dissolved in deionized water (pH ∼ 8–9). Subsequently, the water-immiscible monomer DPA and the initiator either VA-044 (0.41 mg, 0.00125 mmol) and/or V-501 (0.35 mg, 0.00125 mmol) were added to the aqueous solution, maintaining a [CETPA]/[thermal initiator] molar ratio of 1 : 0.25. The amounts of DPA used were as follows: 33 μL for DP_*n*_ 35, 78.9 μL for DP_*n*_ = 85, 158 μL for DP_*n*_ = 170, and 256 μL for DP_*n*_ = 265. The mixtures were purged with argon for 30 minutes. Subsequently, the reaction tubes were immersed in a thermostated oil bath at 45 °C (entries 1–4) and/or 70 °C (entries 5–8), as listed in Table 1, and maintained until the target conversion was reached and a colloidally stable, turbid mixture was formed. Monomer conversion was kinetically followed by taking aliquots from the reaction media and analyzing them by ^1^H NMR spectroscopy. Upon completion, the polymerizations were quenched by immersing the tubes in an ice bath and exposing them to air.

**Table 1:** Experimental and characterization data for the aqueous emulsion RAFT-mediated PISA of PTEGMA_78_-*b*-PDPA_*n*_ diblock copolymer nano-objects, synthesized with varying DPA monomer ratios at different reaction temperatures, at a total solid content of 10 wt%

No.	Target composition	[M]_0_/[CETPA]_0_/[I]_0_	*T* (°C)	Time (h)	Conv.^a^ (%)	*M*_n_,_calc_^b^ (g mol^−1^)	*M*_n_,^c^ (g mol^−1^)	*Đ* d
1	PTEGMA_78_-*b*-PDPA_35_	35/1/0.25	45	10	91	21 200	23 690	1.36
2	PTEGMA_78_-*b*-PDPA_85_	85/1/0.25	45	10	80	24 800	21 050	1.35
3	PTEGMA_78_-*b*-PDPA_170_	170/1/0.25	45	10	70	29 900	19 810	1.39
4	PTEGMA_78_-*b*-PDPA_265_	265/1/0.25	45	10	85	40 525	20 400	1.37
5	PTEGMA_78_-*b*-PDPA_35_	35/1/0.25	70	3	87	21 050	18 760	1.17
6	PTEGMA_78_-*b*-PDPA_85_	85/1/0.25	70	3	83	25 055	29 300	1.14
7	PTEGMA_78_-*b*-PDPA_170_	170/1/0.25	70	3	76	30 920	32 500	1.21
8	PTEGMA_78_-*b*-PDPA_265_	265/1/0.25	70	3	81	39 465	33 430	1.27

a Conversion data determined by ^1^H NMR analysis.

b *M*_n,calc_ = [TEGMA]_0_/[RAFT]_0_ × conv. + *M*_WPTEGMA_ + *M*_CETPA_.

c *M*_n_ and

d *Đ* determined by SEC in DMF as the eluent and poly(methyl methacrylate) (PMMA) as the standard.

## 3. Results and discussion

### 3.1. RAFT emulsion polymerization for PTEGMA_78_-*b*-PDPA_*n*_ diblock copolymer NPs

In the current study, we have highlighted the first evaluation of dual thermo-/pH-responsive PTEGMA_78_-*b*-PDPA_*n*_*in situ* soft matter nanoparticle formation polymerized in water. The process began with the synthesis of a water-soluble, thermo-responsive macromolecular chain transfer agent (mCTA), PTEGMA, *via* RAFT. The polymerization was carried out in DMSO at 70 °C using CETPA as the RAFT agent and AIBN as the thermal initiator. After 16 hours, the polymerization was quenched, achieving a monomer conversion of over 93%, as determined by ^1^H NMR spectroscopy. The DP_*n*_ of the resulting PTEGMA-mCETPA was calculated to be 78, based on the ratio of the integrated vinyl proton peaks of TEGMA (*δ* = 5.23 and 5.75 ppm) to the polymer backbone signals (*δ* = 2.10–1.50 ppm). The DP_*n*_ of about 78 for PTEGMA was carefully chosen to achieve a balanced length—not too long (over 100) and not too short (under 30)—because it is well known that the DP_*n*_ of the macro-CTA plays a crucial role in PISA synthesis. The carefully selected DP_*n*_ ensures enough steric stabilization to prevent nanoparticle aggregation, allows for controlled polymerization, and influences the shape and structure of the NPs formed during the process.^10,47^ This balance enables the formation of well-defined, stable, and stimuli-responsive NPs, making them well suited for applications such as drug delivery.^48^ Following purification, the homopolymer was analyzed by ^1^H NMR spectroscopy (see Fig. S2, in the SI) and by SEC analysis, as shown in Fig. 1C and D (black curves). The SEC results confirmed well-controlled polymerization, indicated by a monomodal molecular weight distribution and a low dispersity value (*Đ* = 1.13). The thermo-responsive PTEGMA_78_-mCETPA was used in the *in situ* aqueous emulsion RAFT-mediated PISA of the pH-responsive DPA monomer at a fixed 10 wt% solid content. This yielded a series of well-defined PTEGMA_78_-*b*-PDPA_*n*_ nanoformulations (entries 1–8, Table 1), varying the PDPA block DP_*n*_ (*n* = 35, 85, 170, 265). The synthetic route is illustrated in Scheme 1.

The PISA process was initially performed at 45 °C, intentionally set below the *T*_cp_ of PTEGMA (entries 1–4, Table 1). Azobis[2-(2-imidazolin-2-yl)propane]dihydrochloride (VA-044) was chosen as the water-soluble radical initiator due to its low decomposition temperature, with a 10-hour half-life at 44 °C in water. Additionally, to explore the PISA process above the LCST-type *T*_cp_ of PTEGMA, polymerization was conducted at around 70 °C using the water-soluble radical initiator 4,4′-azobis(4-cyanovaleric acid) (V-501) (entries 5–8, Table 1). Its higher decomposition temperature enabled reaching these elevated temperatures. Both polymerizations (at 45 °C and 70 °C, see Fig. S3A and B in the SI) were kinetically monitored by NMR spectroscopy to follow DPA monomer conversion during PISA. The target DP_*n*_ for the core-forming PDPA block was 265 in PTEGMA_78_-*b*-PDPA_265_ (entries 4 and 8, Table 1). At various time intervals, 20 μL aliquots of each sample solution were promptly analyzed by ^1^H NMR spectroscopy (Fig. S3A and B in the SI). Monomer conversion was determined by comparing the integrals of the residual vinyl proton signals of DPA at 5.5 and 6.0 ppm with the methylene (CH_2_)_4_ signals from both the DPA monomer and polymer. Since PDPA is insoluble in deuterated water, it does not produce an observable NMR signal. To render the PDPA signals visible, 10 μL of deuterated hydrochloric acid (DCl) was added to protonate the insoluble PDPA.^49,50^ The combined use of D_2_O/DCl ensures minimal solvent proton interference and controls the sample protonation state, allowing more accurate identification and quantification of proton signals in ^1^H NMR spectra. Fig. 1A displays the kinetic plots of ln([M]_0_/[M]_*t*_), revealing a clear increase in the rate of polymerization at 45 °C over time. The polymerization rate of PISA was lower (91% DPA conversion with 10 h), similar to recent findings by Jutta Rieger.^51^ In contrast, the polymerization rate of PISA at 70 °C was much faster. The kinetic plots of ln([M]_0_/[M]_*t*_) (Fig. 1B) exhibit a clear increase in the polymerization rate over time, until the 150 min, followed by a plateau. Monomer conversion reaches approximately 86% after 180 minutes, demonstrating control over the PISA aqueous emulsion polymerization and effective conversion within a relatively short period. Across all eight experimental series, the resulting nanoformulations exhibited no coagulation or sedimentation and consistently achieved high monomer conversions. All NPs were characterized similarly, as shown in Table 1 (entries 1–8). Additionally, the number-average molecular weights (*M*_n_) and dispersity (*Đ*) of all NPs were analyzed by SEC (Fig. 1C and D). Fig. 1C shows the SEC curves for entries 1–4 synthesized at 45 °C. The resulting traces are monomodal and largely overlapping, despite the increasing DP_*n*_ of the second block. This likely reflects the fact that SEC measures the hydrodynamic volume rather than the absolute molecular weight, which can lead to underestimation or minimal apparent differences for complex nanoparticle systems. This effect persists even though DMF, a good solvent for both blocks, was used as the SEC mobile phase.^52–54^ In contrast, entries 5–8 produced by PISA at 70 °C exhibit a clear shift in SEC elution peaks toward higher molecular weights compared to the macro-RAFT agent PTEGMA (black curve), indicating more pronounced chain extension and growth under these conditions (Fig. 1D).

Notably, in many cases, a key factor in determining the morphology of copolymer NPs is the so-called packing parameter *P*.^14,55,56^ The value of the *P* helps predict the self-assembled morphology of copolymer NPs. It is important to note, however, that in the case of *in situ* PISA synthesis, the packing parameter concept cannot yet provide a semi-quantitative understanding of the multiple morphological transformations.^57,58^ The core-forming blocks in the copolymer nano-objects are likely solvated by both the monomer and solvent, but their local concentrations are unknown. This solvation affects the effective volume fraction of the core-forming block, making it difficult to calculate variations in the packing parameter during PISA synthesis.

**Fig. 2:**
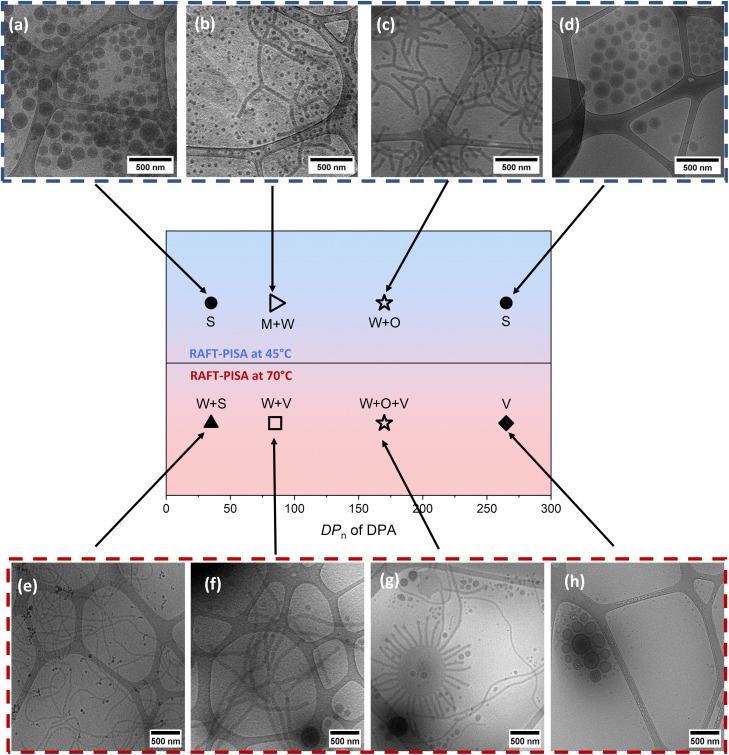
Phase diagram of NPs prepared with varying target DP_*n*_ of the hydrophobic core-forming block *via* aqueous RAFT emulsion polymerization at 45 °C (cryo-TEM images (a)–(d); entries 1–4, in Table 1) and 70 °C (cryo-TEM images (e)–(h); entries 5–8, in Table 1), conducted at 10 wt% solids, along with the corresponding cryo-TEM images. Keys: S = spheres, W + S = worms + spheres, W + O + V = worms + “octopus” + vesicles, V = vesicles, M + W = micelles + worms, and W + V = worms + vesicles.

The resulting PISA nanoformulations were characterized by cryo-TEM, DLS, and SAXS to examine the impact of varying the molar ratio of the core-forming monomer on the particle size and morphology. Samples were diluted with water to approximately 1.0 mg mL^−1^ for cryo-TEM and 0.1 mg mL^−1^ for DLS measurements, which were performed at 25 °C. The phase diagram is shown in Fig. 2, with additional cryo-TEM images of all NPs obtained by RAFT-PISA at 45 °C and 70 °C, respectively. The corresponding light scattering data are provided in Fig. S4A and B in the SI. The physicochemical properties of all NPs (entries 1–8) are summarized in Table 2. For entry 1 in Table 2 (Fig. 2a), which features the shortest PDPA block (DP_*n*_ = 35), spherical nano-objects were observed with diameters generally consistent with the DLS data (see Table 2). The hydrodynamic diameter (*D*_H_) measured by DLS was 219 nm with a relatively broad polydispersity index (PDI) of 0.34 (Fig. S4A, black curve, in the SI). In classical PISA, higher-order morphologies can be achieved by progressively increasing the length of the solvophobic block, provided that chain reorganization is possible. An order–order transition from purely spherical particles to a mixture of small micelles (∼50 nm) and long worm-like structures (∼50 nm in diameter) was observed for entry 2 (Table 2 and Fig. 2b). Indeed, increasing the solvophobic block length induced these higher-order morphologies. DLS measurements for entry 2 showed an apparent *D*_H_ of approximately 72 nm and a relatively narrow size distribution with PDI = 0.21 (Fig. S4A, red curve, in the SI; Table 2).

**Table 2:** Physicochemical characterization of PTEGMA_78_-*b*-PDPA_*n*_, nano-objects obtained *via* a 10-hour and 3-hour aqueous emulsion RAFT/PISA

Entry	Target composition	*D*_H_ a/nm (PDI)	LCST-type phase transitions (°C)		Morph.^b^	*D* c/nm
			*T*_cp_	*T*_CFT_		
1	PTEGMA_78_-*b*-PDPA_35_	219 ± 0.86 (0.34)	38.5	41.5	S	110–150
2	PTEGMA_78_-*b*-PDPA_85_	72 ± 0.35 (0.21)	40.0	43.0	M + W	50 and 50
3	PTEGMA_78_-*b*-PDPA_170_	254 ± 1.15 (0.24)	40.0	43.0	Long W + O	50–60 and 300
4	PTEGMA_78_-*b*-PDPA_265_	330 ± 2.69 (0.37)	38.5	41.5	S	110–180
5	PTEGMA_78_-*b*-PDPA_35_	58 ± 2.92 (0.36)	40.0	43.0	W + S	30
6	PTEGMA_78_-*b*-PDPA_85_	144 ± 0.65 (0.18)	38.5	40.0	W + V	60 and 150–250
7	PTEGMA_78_-*b*-PDPA_170_	397 ± 1.92 (0.24)	38.5	41.5	W + O + V	30, 70–100 and 850
8	PTEGMA_78_-*b*-PDPA_265_	164 ± 0.56 (0.26)	40.0	43.0	V	140–270

a Hydrodynamic diameter and dispersity were determined by DLS at 0.1 wt% in the same solvent used for polymerization, measured at 25 °C.

b Nanoparticle morphology from cryo-TEM (W = worms, M = micelles, O = “octopus”, V = vesicles, and S = spheres).

c Particle diameter from cryo-TEM.

Further increasing the molecular weight of the core-forming block to DP_*n*_ = 170 (entry 3) favored the formation of higher-order morphologies, with a clear transition observed from a mixture of micelles and long worms to a combination of short worms and “octopus-like” structures. Cryo-TEM analysis (Fig. 2c) confirmed the presence of a mixed morphology, featuring flat lamellar disks interconnected by worm-like arms. These “octopus-like” structures exhibited worm arms of approximately 50–60 nm and a central body with a diameter of around 300 nm. It is important to highlight that achieving the intermediate “octopus” morphology as a stable, final structure has long been a significant challenge in RAFT-PISA studies. Traditionally, this complex morphology appears only transiently and is rarely identified as a pure phase in RAFT-PISA phase diagrams. However TEM analyses have occasionally captured such intermediate structures during polymerization in both polar and non-polar media.^59,60^ They have not been reported as the final, stable morphology—until now. Remarkably, in our study, the “octopus” morphology was isolated and confirmed as a distinct, stable phase, marking a noteworthy advancement in the field.

For the longest PDPA block DP_*n*_ = 265 (entry 4, Table 2), well-defined spherical NPs with diameters ranging from about 110 to 150 nm were distinctly observed (Fig. 2d). DLS measurements showed an intensity-average diameter of approximately 330 nm, accompanied by a broader size distribution (PDI = 0.37; Fig. S4A, green curves in the SI; Table 2).

A comprehensive RAFT-PISA study was performed at 70 °C (entries 5–8, Table 2). Surprisingly, for entry 5 in Table 2 (PTEGMA_78_-*b*-PDPA_35_), the cryo-TEM image (Fig. 2e) clearly shows the formation of pure elongated structures (worms) with an estimated diameter of approximately 30 nm along with a small population of spherical particles. Interestingly, despite the shortest PDPA core-forming block, only a very small percentage of spherical morphology was observed—contrary to expectations. This suggests that worm-like morphologies can form at very short core-forming PDPA block lengths under specific conditions—such as high polymerization temperatures—which likely enhance chain mobility and facilitate particle reorganization. For the PTEGMA_78_-*b*-PDPA_85_ nanoformulations, cryo-TEM (Fig. 2f) revealed predominantly worm-like structures with an estimated diameter of approximately 60 nm accompanied by a minor population of vesicles ranging from 150 to 250 nm in size. At a PDPA block length of DP_*n*_ = 170, cryo-TEM (Fig. 2g) revealed intricate, highly branched “octopus-like” aggregates. These structures feature a central flat bilayer from which symmetrically arranged cylindrical arms extend—each measuring approximately 850 nm—accompanied by smaller spherical particles around 70–100 nm in diameter. This unique intermediate morphology appears to represent a dynamic transition phase, bridging worm-like assemblies and fully formed vesicles as the hydrophobic block length increases. It is important to note that the “octopus” morphology consistently emerges at this block ratio at RAFT-PISA synthesis temperatures of both 45 °C and 70 °C, highlighting the robustness of its formation across varying conditions. Probably, this is attributed to sufficient core plasticization and chain mobility at both temperatures, combined with polymer block length and system conditions that create a stable intermediate morphology window across this temperature range.^10^

To form vesicular NPs, it is important to have a core-forming block with a sufficiently high DP, while keeping the stabilizer block DP relatively short. As anticipated, further extending the PDPA block to DP_*n*_ = 265 led to the formation of exclusively vesicular structures, as confirmed by cryo-TEM analysis (Fig. 2h). For the PTEGMA_78_-*b*-PDPA_265_ NPs, DLS analysis showed an average diameter of approximately 164 nm with a broad size distribution (PDI = 0.26) (Fig. S4B, green DLS curves in the SI; Table 2).

Cryogenic TEM confirmed the presence of diverse nanoparticle morphologies in solution, supporting the morphological transformations driven by increasing the DP_*n*_ of the hydrophobic PDPA block. Moreover, the formation of higher-order morphologies in dual-responsive NPs was strongly dependent on the assembly pathway, especially under different temperature conditions. Utilizing PTEGMA as a macro-chain transfer agent facilitated the generation of a variety of structures—including micelles, worms, mixed worm–vesicle systems, distinct “octopus”-like assemblies, and pure vesicles. This structural diversity reflects the complex interplay between hydrophilic and hydrophobic segments, demonstrating how subtle changes in block length and composition govern self-assembly behavior and final morphology.

**Fig. 3:**
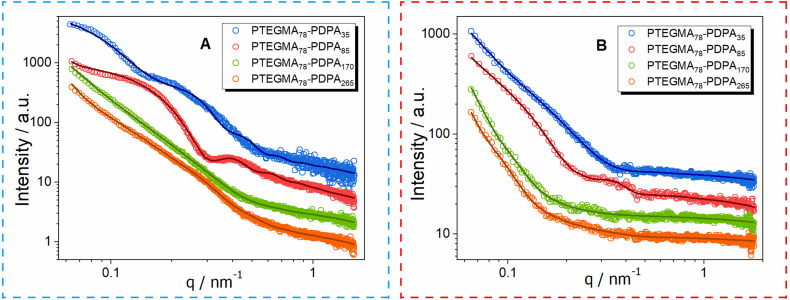
Fitted SAXS curves of PTEGMA_78_-*b*-PDPA_*n*_ prepared *via* aqueous emulsion RAFT/PISA at (A) 45 °C or (B) 70 °C.

To validate these findings, SAXS measurements were carried out at 25 °C for all NPs (entries 1–8, see Fig. 3A and B), with the results summarized in Table 3. The scattering profiles were analyzed using SASfit software (version 0.94.10), enabling effective data fitting. A combination of two models was employed to obtain satisfactory fits: the core–shell model^61^ for spherical and vesicular particles, and the self-avoiding flexible cylinder model^62^ for worm-like and octopus morphologies. In the core–shell model, the “core” typically represents the dense polymeric region, while the “shell” corresponds to a less dense or more solvated layer—this may include solvent molecules or distinct polymer segments that contribute to particle stability and solvent interactions. During the fitting process, parameters associated with different structural morphologies were adjusted alternately to refine the model. Particle polydispersity was incorporated into the form factors using a log-normal distribution, providing a more accurate description of the size distribution within the samples. The solvent effect (water) was approximated by a constant background function. A detailed description of the fitting models is provided in the SAXS chapter of the SI, and the parameters for all analyzed samples are summarized in Table 3. For clarity, parameters related to the self-avoiding flexible cylinder model carry a subscript w. Polydispersity factors for both the core–shell core radius and the flexible cylinder cross-section radius are also included. In cases where multiple models were applied, individual model contributions to the total fit are presented in Fig. S5 in the SI.

**Table 3:** Parameters of the core–shell and self-avoiding flexible cylinder models for SAXS data fitting for PTEGMA_78_-*b*-PDPA_*n*_ NPs including polydispersity ratios for shell and cross-section radii^a^

	Samples prepared at reaction temperature *T* = 45 °C				Samples prepared at reaction temperature *T* = 70 °C			
Entry	1	2	3	4	5	6	7	8
Target composition	PTEGMA_78_-*b*-PDPA_35_	PTEGMA_78_-*b*-PDPA_85_	PTEGMA_78_-*b*-PDPA_170_	PTEGMA_78_-*b*-PDPA_265_	PTEGMA_78_-*b*-PDPA_35_	PTEGMA_78_-*b*-PDPA_85_	PTEGMA_78_-*b*-PDPA_170_	PTEGMA_78_-*b*-PDPA_265_
*R*_core_/nm	36.5 ± 1.9	32.8 ± 1.6	83.1 ± 3.9	138 ± 14	—	49.1 ± 1.6	87.5 ± 4.4	147 ± 13
PD (*R*_core_)	0.49 ± 0.02	0.79 ± 0.03	0.58 ± 0.03	0.59 ± 0.09	—	0.18 ± 0.03	0.25 ± 0.01	0.23 ± 0.06
*R*_shell_/nm	27.6 ± 0.7	13.9 ± 2.1	8.81 ± 0.15	25.3 ± 1.5	—	8.76 ± 0.42	9.15 ± 0.11	7.42 ± 0.23
Δ*η*_core_/Å^−2^	(6.10 ± 0.14) × 10^—3^	(6.40 ± 0.52) × 10^−2^	(2.27 ± 0.29) × 10^—4^	(8.95 ± 0.69) × 10^−3^	—	(5.49 ± 0.31) × 10^−4^	(3.54 ± 0.33) × 10^−4^	(3.28 ± 0.54) × 10^−4^
Δ*η*_shell_/Å^−2^	(1.06 ± 0.11) × 10^—4^	(2.60 ± 0.11) × 10^−4^	(3.16 ± 0.18) × 10^—5^	(4.29 ± 0.48) × 10^−5^	—	(1.12 ± 0.06) × 10^−5^	(6.56 ± 0.18) × 10^−5^	(1.95 ± 0.36) × 10^−5^
*R*_w_/nm	—	31 ± 2	45 ± 3	—	25 ± 3	48 ± 3	94 ± 6	—
PD (*R*_w_)	—	0.35 ± 0.06	0.68 ± 0.05	—	0.19 ± 0.09	0.27 ± 0.02	0.37 ± 0.04	—
*L*_w_/nm	—	638 ± 83	1551 ± 109	—	2915 ± 265	1726 ± 154	2324 ± 162	—
*b*_w_/nm	—	226 ± 49	390 ± 41	—	38 ± 21	125 ± 43	488 ± 59	—
*L*/*b* ratio	—	3	4	—	77	14	5	—

a Parameters with a lower index ‘core’ or ‘shell’ belong to the core–shell model, parameters with a lower index ‘w’ belong to the self-avoiding flexible cylinder model.

Both PTEGMA_78_-*b*-PDPA_35_ NPs (entries 1 and 4, Table 3; blue curves in Fig. 3A and B) exhibited simple morphologies. Spherical particles with an average radius of 64.1 nm and moderate polydispersity were observed for entry 1, consistent with the TEM image (see Fig. 2a). In contrast, for entry 5, exclusively worm-like structures were observed characterized by an average cross-sectional radius of 25 nm and a length-to-breadth (*L*/*b*) ratio of approximately 77, indicating a high degree of flexibility. The TEM image (Fig. 2e) confirms the presence of only worm-like particles; however, their flexibility does not appear to be substantially greater than that observed in other samples with similar structures. This apparent enhanced flexibility may result from the higher concentration of worm-like particles in this sample, which, being the sole morphology present, increases overlap and creates the illusion of greater flexibility. Notably, this sample lacks any spherical particles.

The PTEGMA_78_-*b*-PDPA_85_ nano-objects (entries 2 and 6, Table 3; red curves in Fig. 3A and B) contained a more complicated morphology with both spherical and worm-like particles, as can be seen from the TEM images (Fig. 2b and f). SAXS experiments showed strong scattering contrast for both samples, revealing distinct features in the curves. Curve fitting of entry 2 indicated the presence of highly polydisperse spherical particles with an average radius of 46.7 nm and worm-like structures with a cross-sectional radius of 31 nm and an *L*/*b* ratio of 3, indicating very stiff worms. Entry 6 showed similar particles, but with a slightly bigger radius (57.9 nm for spherical particles and 48 nm for worm-like particles) and a lower polydispersity. The *L*/*b* ratio of the worm-like particles also increased to 14, indicating substantially higher flexibility. However, this flexibility might be somewhat overestimated due to particle overlap at the elevated worm concentration.

The PTEGMA_78_-*b*-PDPA_170_ nanoformulations (entries 3 and 7, Table 3; green curves in Fig. 3A and B) exhibited highly complex morphologies. TEM images (Fig. 2c and g) revealed a mixture of vesicles, worm-like particles, and distinctive “octopus” structures. This complexity complicates SAXS data interpretation. Nonetheless, SAXS curves were fitted using a combined model incorporating core–shell particles and self-avoiding flexible cylinders. It is important to note that the “body” of the octopus exceeds the SAXS detection range, so only the “tentacles” were modeled alongside worm-like structures using the flexible cylinder model. The SAXS fitting parameters were relatively consistent across both temperature conditions. Vesicular particles exhibited moderate polydispersity, low scattering contrast, and average radii between 90 and 100 nm. Worm-like particles displayed a low *L*/*b* ratio, suggesting limited flexibility, possibly influenced by the rigid octopod tentacles observed in TEM images. Key differences between entries 3 and 7 included a larger cross-sectional radius of worm-like particles obtained at a polymerization temperature of 70 °C (entry 7) and shifts in the relative abundance of morphologies. Entry 3 contained a higher proportion of worm-like particles, whereas entry 7 had a greater presence of core–shell vesicles. This variation is reflected in the SAXS intensity profiles, where the dominant particle type corresponds to higher model intensity, as shown in Fig. S5B and S6D in the SI.

The morphology of the PTEGMA_78_-*b*-PDPA_265_ NPs was notably simpler, consisting exclusively of vesicular particles (entries 4 and 8; Table 3; orange curves in Fig. 3A and B). SAXS curve fitting with the core–shell model yielded vesicle parameters with total radii exceeding 150 nm, approaching the upper detection limit of SAXS and thus increasing measurement uncertainty. Despite this, discernible differences between entries 4 and 8 were observed: entry 4 exhibited higher scattering contrast and greater polydispersity—consistent with trends seen in other lower-temperature samples—compared to entry 8. Additionally, the vesicle shell thickness in entry 4 was significantly greater than that observed in entry 8, corroborated by the TEM images (Fig. 2d and h).

### 3.2. Thermo- and pH-responsive behavior of the nano-objects

**Fig. 4:**
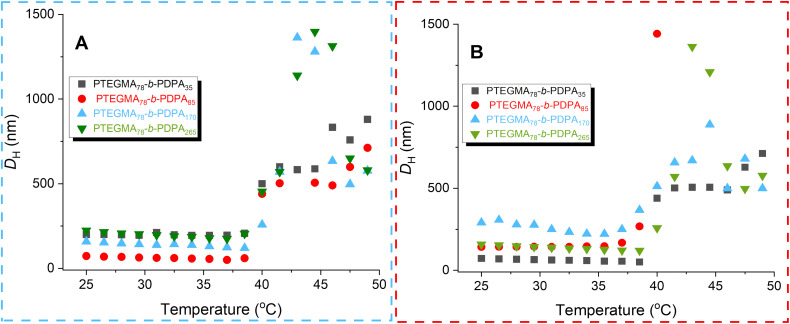
Temperature dependence of the *D*_H_ of PTEGMA_78_-*b*-PDPA_*n*_ nano-objects: (A) entries 1–4 and (B) entries 5–8 (Table 2).

As mentioned above, the PTEGMA_78_-*b*-PDPA_*n*_ diblock copolymer NPs are engineered to respond precisely and reversibly to simultaneous changes in temperature and pH, allowing controlled modulation of their properties, structure, and behavior for advanced, tunable applications. These nano-objects are expected to exhibit thermo-responsive behavior due to the shell-forming PTEGMA block. The LCST-like transition, including the cloud point temperature (*T*_cp_) and phase separation behavior (*T*_CFT_) of all nano-objects (entries 1–8, Table 2), was investigated using variable-temperature DLS analysis at a concentration of 0.1 mg mL^−1^. The measurements were conducted over a temperature range of 25 to 50 °C, with increments of 1.5 °C. Fig. 4A and B show the temperature dependence of the *Z*-average *D*_H_.

All the nano-objects exhibit a similar pattern, with a sharp increase in size from nanometers to several micrometers once the *T*_cp_ is reached (see Fig. 4A and B). It is important to note that the absolute size measured above the *T*_cp_ should be interpreted with caution, as gravitational sedimentation of larger aggregates can occur during the analysis time at this scale. What remains reliable, however, is the dynamics of phase separation driven by the loss of steric stabilization. Specifically, once the *T*_cp_ is exceeded, the thermoresponsive shell (PTEGMA) collapses onto the nanoparticle core, resulting in aggregation. It is worth noting that the *T*_cp_ is independent from targeted different chain lengths for all the average degree of polymerization considered.^63^ The *T*_CFT_ corresponds to the *D*_H_ value at the onset of the rapid particle size increase. Table 2 lists the *T*_cp_ and *T*_CFT_ values for entries 1–8. Fig. 4A and B clearly show that this series of nano-objects (entries 1–8) exhibits thermoresponsive behavior within a narrow cloud point range of 38.5 °C to 41.5 °C.

**Fig. 5:**
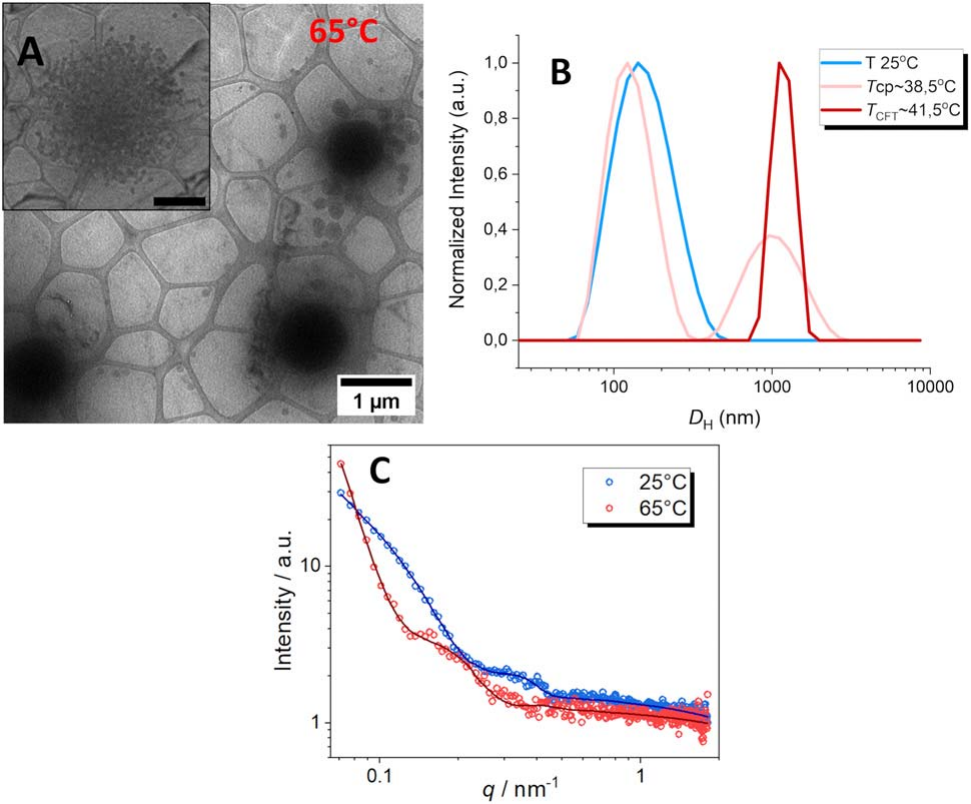
(A) Representative cryo-TEM image of PTEGMA_78_-*b*-PDPA_85_ (entry 6) nano-objects at 65 °C. The scale bars in the zoomed cryo-TEM image represent 1 μm; (B) DLS plot for the *D*_H_ at *T* = 25 °C (blue curve), *T*_cp_ 38.5 °C (light pink curve) and at the *T*_CFT_ = 41.5 °C (see the dark red curve); (C) SAXS profile at 25 °C (in blue) and 65 °C (in red).

To further investigate and enhance the temperature-responsive behavior of these NPs, we characterized a heated sample using cryo-TEM, DLS and SAXS. The PTEGMA_78_-*b*-PDPA_85_ formulation (entry 6) was chosen as a representative example due to its complex morphology, as illustrated in Fig. 2f. For the cryo-TEM analysis, the sample was heated at 65 °C overnight in an oven and then rapidly deposited onto cryo-TEM grids, following a protocol described in published work.^64^ The above cryo-TEM image of entry 6 at 25 °C (below the *T*_cp_) reveals well-defined worm-like structures, accompanied by a minor population of both small and large spherical particles (see Fig. 2f). Upon heating to 65 °C (above the *T*_cp_), the morphology underwent a significant transformation, shifting to large spherical aggregates, as shown in Fig. 5A. This observation demonstrates that heating induces considerable changes in both morphology and size, which can be attributed to the transition of the PTEGMA chains from a hydrophilic to a hydrophobic state. Furthermore, it is well established that the LCST represents the lowest temperature at which phase separation occurs. Above this phase boundary, polymer chains tend to collapse or undergo aggregation, leading to particle size changes.^65^ Indeed, the DLS results corroborate the TEM findings, providing complementary evidence for the temperature-dependent structural evolution of the NPs. Fig. 5B shows the DLS measurements of entry 6 at 25 °C (blue curve), at a temperature above the *T*_cp_ 38.5 °C, (light pink curve) and at the *T*_CFT_ = 41.5 °C (see the dark red curve), respectively. Above *T*_CFT_ (41.5 °C), the sample became highly turbid, and the DLS data exhibited multiple scattering effects, indicating the formation of large aggregates that sediment, leading to unstable and inconsistent DLS results, as observed in Fig. 4A and B.

The thermally induced transition from worms-like structures to micelle aggregation was confirmed using characteristic SAXS patterns recorded at different temperatures, specifically 25 °C and 65 °C, as shown in Fig. 5C. At 65 °C, worm-like particles were no longer observed, while in the core–shell, the particle size increased significantly compared to 25 °C, accompanied by an increase in polydispersity and a noticeable decrease in scattering intensity. The larger particle size and higher polydispersity are characteristic of aggregation, while the reduced intensity can be attributed to sedimentation. These observations are consistent with the results obtained from both cryo-TEM and DLS analyses.

**Fig. 6:**
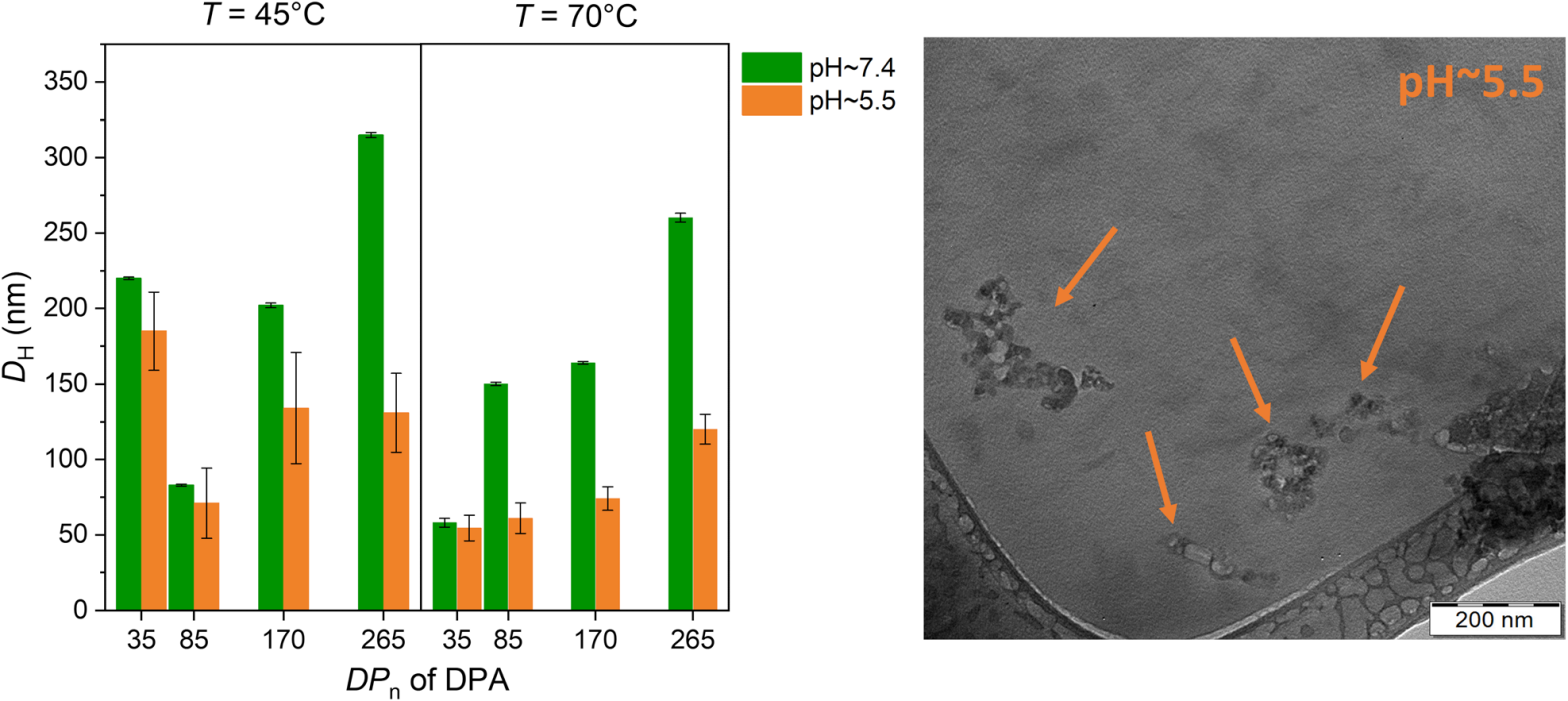
Representative hydrodynamic diameters of PTEGMA_78_-*b*-PDPA_*n*_ NPs synthesized at two different polymerization temperatures (45 °C and 70 °C), plotted as a function of the DP_*n*_ of the PDPA block (left). Green and orange bars represent measurements at pH ∼7.4 and pH ∼5.5, respectively. Cryo-TEM images of PTEGMA_78_-*b*-PDPA_85_ (entry 6, in Table 2) upon contact under acidic conditions (pH ∼ 5.5; right). The arrows depict structurally disassembled NPs.

Additionally, the NPs are expected to exhibit pH-responsive behavior due to the pH sensitivity of the PDPA block, which has a p*K*_a_ of approximately 6.2–6.3. All NPs (entries 1–8) were prepared to mimic physiological conditions before being adjusted to either PBS buffer at pH 7.4 (reflecting blood plasma) or acetate buffer at pH 5.5 (representing endosomal conditions after cellular internalization) prior to DLS analysis. Measurements were conducted at ambient temperature (∼25 °C) to assess the combined effect of polymer chain length—expressed as the DP_*n*_ and solution pH on nanoparticle size. It should be noted that the intensity-average *D*_H_ is calculated using the Stokes–Einstein equation (eqn (1), SI), thus reporting an apparent ‘sphere-equivalent’ size for non-spherical particles. The left panel of Fig. 6 shows a systematic study of the average particle size at both pH values as a function of the DP_*n*_ of the PDPA block. At neutral pH (∼7.4), the PDPA block remains predominantly hydrophobic, which favors the formation of well-defined NPs with relatively larger diameters. In contrast, at acidic pH (∼5.5), protonation of the DPA units increases their hydrophilicity, causing partial swelling or disassembly of the nanoparticles.^44,66^ This leads to a noticeable decrease in particle size and/or broader size distributions, clearly demonstrating the pH-responsive nature of the system. The PTEGMA_78_-*b*-PDPA_85_ formulation (entry 6 in Table 2) was examined by cryo-TEM to investigate potential morphological changes induced by an acidic environment (pH ∼5.5). Visual analysis of the cryo-TEM images, shown in Fig. 6 (right panel), clearly demonstrates that lowering the pH to ∼5.5 triggers significant structural disassembly of the nano-objects. This disintegration highlights the pH-responsive nature of the NPs, likely due to protonation of the PDPA block under acidic conditions, which increases hydrophilicity and destabilizes the core, leading to the breakdown of the previously well-defined nanostructures.

Our findings confirm that at physiological pH, larger and more stable NPs form due to hydrophobic interactions, whereas acidic conditions induce protonation of the PDPA block, resulting in size reduction. These insights are essential for designing nanoformulations with tunable properties for applications such as targeted drug delivery, where precise control over particle size and responsiveness to the tumor microenvironment is critical.

## 4. Conclusions

In summary, the PISA of the pH-responsive DPA monomer *via* aqueous emulsion RAFT polymerization, using the thermoresponsive PTEGMA_78_-mCETPA as a macromolecular chain transfer agent, enables the *in situ* formation of novel dual-responsive soft-matter nano-objects. While the hydrophilic PTEGMA block length was held constant, the hydrophobic PDPA block length was systematically varied (DP_*n*_ = 35–265) to explore its influence on nanoparticle morphology. The PISA process was carried out at two distinct temperatures, below and above the LCST of PTEGMA, namely 45 °C and 70 °C, at 10 wt% solids in aqueous media, achieving high monomer conversion rates. Our study demonstrates a pathway-dependent formation of higher-order morphologies in these dual-responsive NPs by finely tuning the hydrophilic-to-hydrophobic block ratio. The polymerization conditions significantly influenced the resulting nanoparticle structures, yielding a diverse spectrum of morphologies. At 45 °C, predominantly spherical nano-objects were formed for most compositions, except for PTEGMA_78_-*b*-PDPA_85_ and PTEGMA_78_-*b*-PDPA_170_, which exhibited a mixture of spherical, worm-like, and more complex “octopus”-like architectures. In contrast, polymerization at 70 °C favored the emergence of worm-like, “octopus,” and vesicular structures. This morphological diversity arises from the delicate interplay between the hydrophilic PTEGMA and hydrophobic PDPA blocks, which governs the self-assembly pathways and stabilizes distinct nanostructures. The dual thermo- and pH-responsive behavior of these nano-objects, revealing an LCST-type phase transition upon heating and nanoparticle disassembly under acidic conditions, was studied. This work significantly advances polymer science by clarifying how block copolymer composition influences self-assembly and nanoparticle morphology. Systematic tuning of block lengths enables precise control over the architecture and allows for on-demand adjustment of material properties.

## Author contributions

S. L. P. synthesized the novel dual-responsive soft matter NPs, performed the DLS experiments, analyzed the data and wrote the manuscript. E. P. performed cryo-TEM experiments and analyzed the data. M. H. provided financial support. V. P. performed SAXS experiments and analyzed the data. All authors have given approval to the final version of the manuscript.

## Conflicts of interest

There are no conflicts to declare.

## Supplementary Material

NA-OLF-D5NA00779H-s001

## Data Availability

Data for this article, including NMR, SEC, DLS, cryo-TEM and SAXS raw data, are available at the ASEP repository at https://asep-portal.lib.cas.cz. Supplementary information (SI) includes experimental details, ^1^H NMR, SEC, DLS, cryo-TEM, and SAXS analyses; it also contains details on the synthesis of 4-cyano-4-(((ethylthio)carbonothioyl)thio)pentanoic acid (CETPA); ^1^H NMR spectrum of CETPA and PTEGMA, ^1^H NMR spectra from RAFT-PISA kinetics; DLS size distributions for PTEGMA_78_-*b*-PDPA_*n*_ (*n* = 35–265) nanoparticles; and SAXS model fitting results. See DOI: https://doi.org/10.1039/d5na00779h.
