# Supplementary material for: Synthesis of morphologically diverse dual thermo- and pH-responsive nano-objects via RAFT-mediated emulsion PISA
Source: Nanoscale Adv. 2025 Oct 10;7(24):8023–36. doi: 10.1039/d5na00779h (PMC12584748; doi:10.1039/d5na00779h)
Supplement: NA-007-D5NA00779H-s001 [file NA-007-D5NA00779H-s001.pdf]

Supplementary Information (SI) for Nanoscale Advances.
This journal is © The Royal Society of Chemistry 2025

Supporting Information for the Manuscript Entitled

Synthesis of Morphologically Diverse Dual Thermo- and pH-Responsive Nano-Objects *via* RAFT-Mediated Emulsion PISA

Svetlana Lukáš Petrova, Ewa Pavlova, Martin Hrubý and Václav Pokorný*

Institute of Macromolecular Chemistry v.v.i., Academy of Sciences of the Czech Republic, Heyrovsky Sq. 2, 162 06 Prague 6, Czech Republic

*Correspondence: petrova@imc.cas.cz; Tel: +420 296 809 296

Instruments and Analyses:

Proton nuclear magnetic resonance (^{1}H NMR) spectroscopy. The CETPA, PTEGMA$_{78}$-mCETPA and PTEGMA-*b*-PDPA NPs were characterized by ^{1}H NMR spectroscopy. ^{1}H NMR spectra were recorded on a Bruker Avance III 400 spectrometer operating at 400 MHz, using $CDCl_3$ and D_2O + 10μL as the solvents at 295 K and 32 scans were applied.

Size exclusion chromatography (SEC). The SEC analyses were performed using a Deltachrom SDS 030 pump (Watrex Ltd., Prague, Czech Republic) with a flow rate of 0.5 ml/min and an autosampler MIDAS (Spark Ltd., Emmen, Holland). Two PLgel 10-µm mixed B LS columns (Polymer Laboratories, Shropshire, UK) were used in a series, each separating according to the producer in the molecular weight range of approximately $5 \times 10^2 \leq M \leq 1 \times 10^7$(related to PS standards). UV/VIS DeltaChrom UVD 200 detector (Watrex) with a flow cell volume of 8 µl, operating at a wavelength λ=264 nm. The measurements were conducted in DMF (Honeywell, Riedel-de-Haën, Germany, for HPLC ≥ 99.9%,) with 50 mM LiBr as the mobile phases, at a flow rate of 1mL/min and ambient temperature. For the determination of M_w and M_n, a calibration curve was constructed using poly(methyl methacrylate) (PMMA) standards. Data were collected using Clarity software (DataApex Ltd.).

The dynamic light scattering (DLS). The NPs of PTEGMA-*b*-PDPA obtained from the reaction mixture were diluted to 0.1 mg·mL^{-1} by water (pH 8-9) and measurements were performed using an ALV CGE laser goniometer consisting of a 22 mW HeNe linear polarized laser operating at a wavelength (λ = 632.8 nm), an ALV 6010 correlator, and a pair of avalanche photodiodes operating in pseudo-cross-correlation mode. The samples were loaded into 10 mm diameter glass cells and maintained at 25 °C. The data were collected using the ALV Correlator

Control software and the counting time was 45 s and was repeated three times. The instrument software was used to calculate the average hydrodynamic diameter D_H and the width of the distribution denoted as the polydispersity index PDI. The measured intensity correlation functions $g_2(t)$ were analyzed using the algorithm REPES (incorporated in the GENDIST program), resulting in the distributions of relaxation times shown in equal area representation as $\tau A(\tau)$. The mean relaxation time or relaxation frequency ($\Gamma = \tau^{-1}$) is related to the diffusion coefficient (D) of the nanoparticles as $D = \frac{\Gamma}{q^2}$ where $q = \frac{4\pi n \sin\frac{\theta}{2}}{\lambda}$ is the scattering vector being n the refractive index of the solvent and θ the scattering angle. The hydrodynamic radius (R_H) or the distributions of R_H were calculated by using the Stokes-Einstein relation:

$$R_H = \frac{k_B T q^2}{6\pi\eta D}\tau \quad (1)$$

where k_B kis the Boltzmann constant, T the absolute temperature, and η the viscosity of the solvent.

Small-angle X-ray scattering (SAXS). The SAXS profiles were recorded by two-pinhole point focusing SAXS instrument Xeuss Pro made by Xenocs (Grenoble, France). The CuKα beam with a wavelength of λ=0.154 nm was generated by a low-power Genix3D microsource operating at 50 kV and 0.6 mA. The whole system was evacuated and scattering was observed with a windowless 2D Eiger2 R 1M detector (Dectris; Baden-Dättwil, Switzerland). Sample to detector distance was set to 1000 mm and the exposure time to 30 minutes for each sample.

In the core-shell model, the scattering intensity is defined by the following equation:

$$I(q) = [K\ (q,Rcore,\Delta\eta core) + K(q,Rcore + \Delta Rshell,\Delta\eta shell) - K(q,Rcore,\Delta\eta shell - \Delta\eta cor \text{[illegible]} \; background \quad (2)$$

This equation describes how the scattering intensity varies with the scattering vector, providing insights into the structural characteristics of the nano-objects with

$$K(q,R,\Delta\eta) = \frac{4}{3}\pi R^3 \Delta\eta 3 \frac{\sin(qR) - qR\cos(qR)}{(qR)^3} \quad (3)$$

where R_{core} is the radius of the core, ΔR_{shell} is the shell thickness, and $\Delta\eta$ is the scattering length density difference between core/shell and the solvent.

The log-normal distribution is defined by equation 4:

$$f(x) = \frac{1}{Norm} \frac{1}{x\sigma} \exp\left(- \frac{1}{2}\left(\frac{\ln(x) - \mu}{\sigma}\right)^2 \right) \tag{4}$$

where Norm is a normalization factor determined during numerical calculation, μ=ln(x_{med}), x_{med} is the median value of the distribution and σ (=PD or polydispersity ratio) is a parameter describing the width of the underlying normal distribution.

The self-avoiding flexible cylinders (WormLikeChainEXV) model is characterized by the structure factor $F_S(q,R,L)$ which consists of a cross-section term $F_{cs}(q,R)$ and a longitudinal term $F_L(q,L)$, so that $F_S = F_{cs} \cdot F_L$ with

$$F_{cs}(q,R) = \left[\frac{2B_1(qR)}{qR}\right]^2 \tag{5}$$

where $B_1(x)$ is the first order Bessel function of the first kind and

$$F_L(q,L) = 2Si(qL) / (qL) - 4\sin^2(qL / 2) / \left(q^2L^2\right) \tag{6}$$

where

$$Si(x) = \int_0^x t^{-1}\sin t\,dt \tag{7}$$

The parameter R describes the cross-section radius of the worm, L is the total length of the worm and b is the Kuhn length which describes the stiffness of the chain and corresponds to twice the length of locally stiff segments. The exponents of the Bessel function is strongly dependent on the L/b ratio and the behavior is quite complex, so we refer the reader to the original text for details.

Cryogenic Transmission Electron Microscopy Analyses (cryo-TEM). Cryo-TEM observations were performed through a Tecnai G2 Spirit Twin 12 (FEI, Czech Republic), equipped with cryo-attachment (Gatan, cryo-specimen holder) using a bright field imaging mode at an accelerating voltage of 120 kV.

Polymer solutions were prepared at 1 wt% in ultra-pure water. Typically, 4 µL of the sample solution was deposited on an electron microscopy grid covered with lacey carbon supporting film (Agar Scientific) after hydrophilization by glow discharge. The solution excess was removed by blotting (Whatman no. 1 filter paper) for ~1s and then the grid was immediately plunged into liquid ethane held at -181 C. The frozen sample was transferred into the

microscope and observed at -173 C under the conditions described above (120 kV, bright field imaging).

Synthesis of 4-cyano-4-(((ethylthio)carbonothioyl)thio)pentanoic acid (CETPA). Sodium hydride (60 % in mineral oil, 3.15 g, 0.079 mol) was added to diethyl ether (150 mL) in a 250 mL round bottom flask and was cooled to <10 °C. Ethanethiol (4.72 g, 0.076 mol) was added dropwise (over 20 minutes) to the suspension containing sodium hydride and the resultant mixture was allowed to stir for 10 minutes. To this solution, carbon disulfide (6 g, 0.079 mol was then added dropwise. After a further 10 minutes, the resultant ethane trithiocarbonate sodium salt (7.0 g, 0.044 mol) was isolated by filtration by suction using a fritted glass funnel. The ethane trithiocarbonate sodium salt was resuspended in diethyl ether (100 mL) and iodine (5.6 g, 0.022 mol) was added and stirred for 45 minutes at room temperature. Subsequently, the brown suspension was washed twice with $Na_2S_2O_3$ (200 mL of 5 wt. %) solution and once with brine (200 mL) to yield a yellow ether phase which was collected, dried over $MgSO_4$, filtered and concentrated via rotary evaporation to yield a viscous yellow liquid (3.07 g, 0.011 (mol). ^{1}H-NMR (400 MHz, $CDCl_3$ δ, ppm) 3.30 (q, 4H), 1.34 (t, 6H).

The bis(ethyl trithiocarbonate) was dissolved in ethyl acetate (100 mL) and 4,4'-azobis(4-cyanopentanoic acid) (V-501, 4.57 g, 0.0169 mol) was added. This solution was refluxed under nitrogen at 90-100 °C for 20 hours. The solution was concentrated and purified by flash silica chromatography (30.0 × 5.0 cm) using n-pentane/ethyl acetate/acetic acid (1/1/0.03 v/v/v). After rotary evaporation of the appropriate fractions (Rf = 0.65 in n-pentane/ethyl acetate/acetic acid 1/1/0.03 v/v/v), a viscous yellow liquid was obtained. The yellow liquid was placed at -20 °C, which yielded yellow crystals that were subsequently filtered, washed with ice-cold pentane and dried in vacuo. Yield: 5.76 g.

^{1}H-NMR (400 MHz, $CDCl_3$, δ, ppm): 3.33 (q, 2H, CH_2CH_3), 2.67 (m, 2H, CH_2CH_2), 2.52 (m, 1H, CH_2CH_2), 2.39 (m, 1H, CH_2CH_2), 1.86 (s, 3H, CH_3), 1.34 (t, 3H, CH_3). Conversion by ^{1}H-NMR spectroscopy 85% (See Figure S1 for assignments).

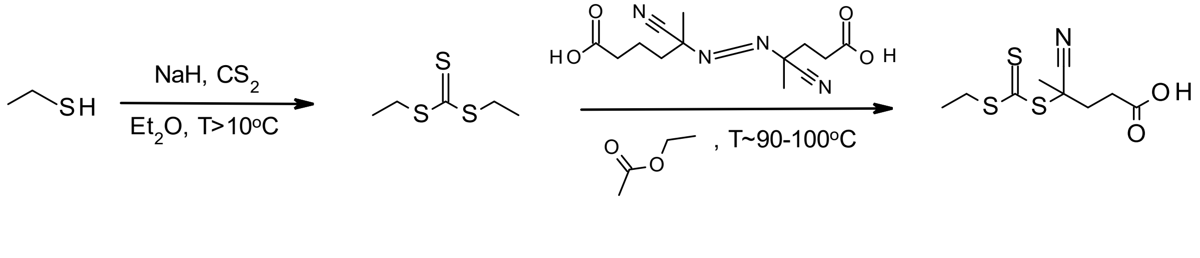

Scheme S1. Schematic representation of the synthesis of 4-cyano-4-(((ethylthio)carbonothioyl)thio)pentanoic acid (CETPA).

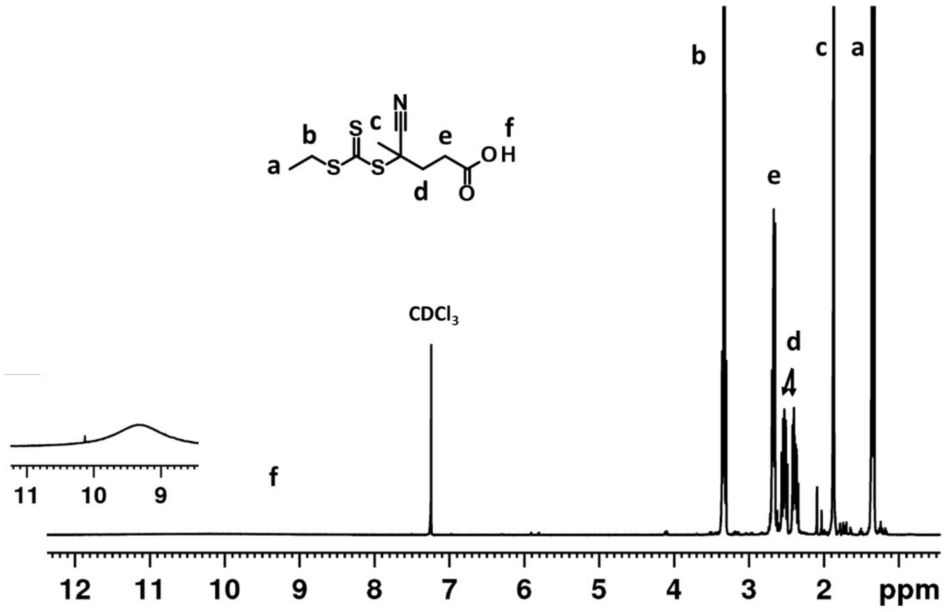

Figure S1. ^{1}H NMR spectrum of 4-cyano-4-(((ethylthio)carbonothioyl)thio)pentanoic acid (CETPA) in $CDCl_3$.

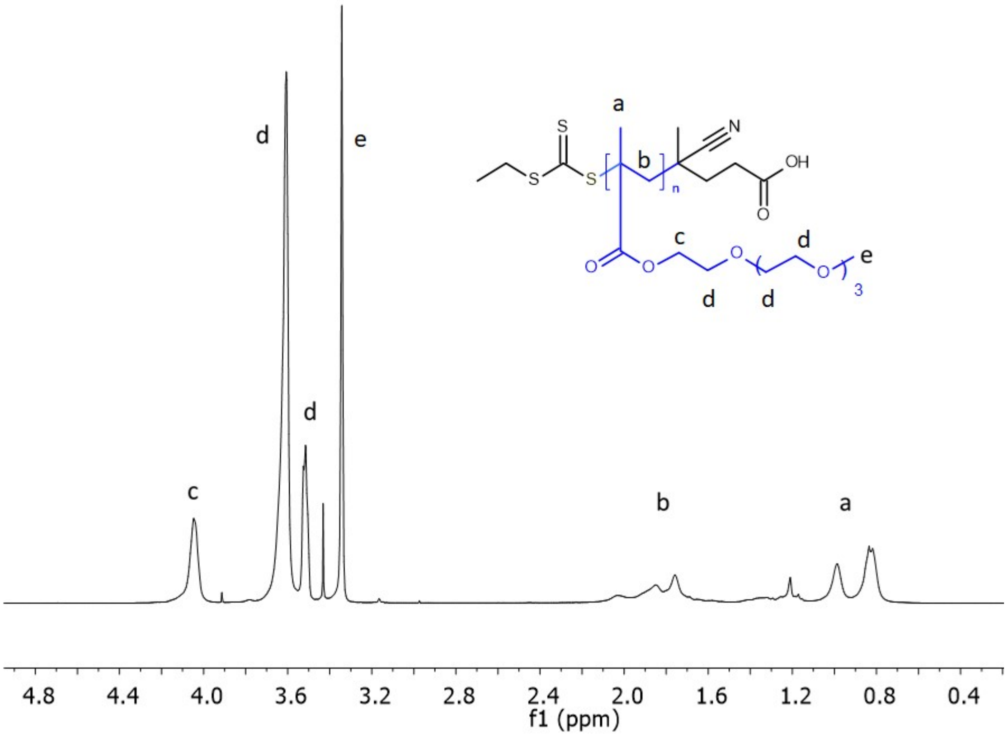

Figure S2. ^{1}H NMR spectrum of the $PTEGMA_{78}$-mCETPA in $CDCl_3$.

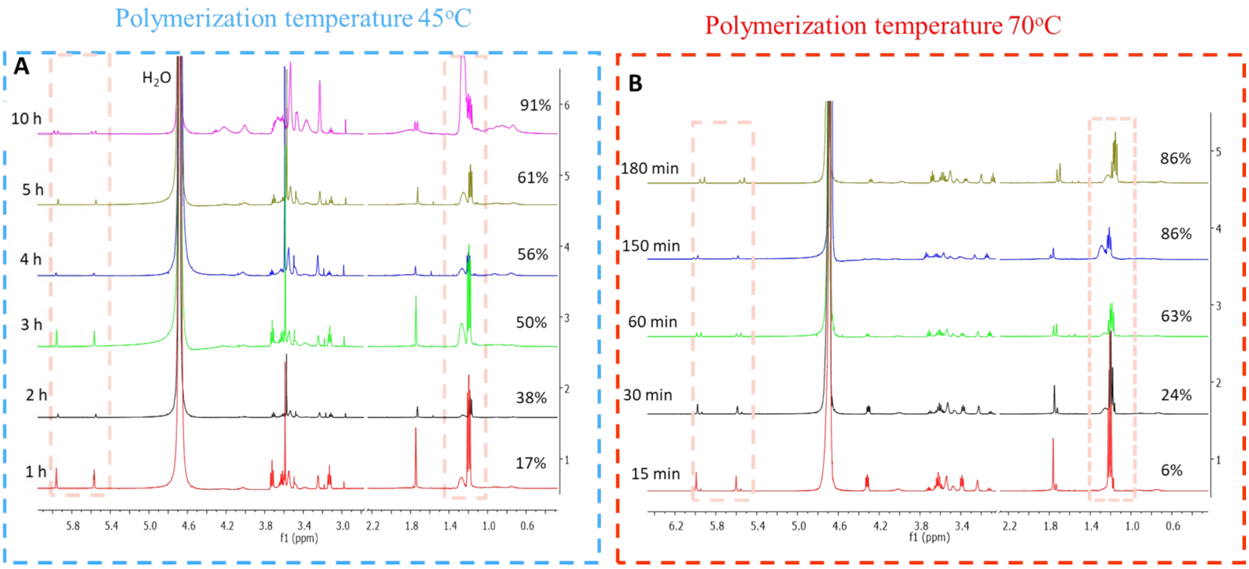

Figure S3. ^{1}H NMR spectra recorded during the kinetic screening of RAFT-PISA for PTEGMA$_{78}$-*b*-PDPA$_{265}$ (entries 4 and 8, Table 1) at two polymerization temperature of 45 °C (**A**) and 70 °C (**B**) in D_2O + 10µL DCl.

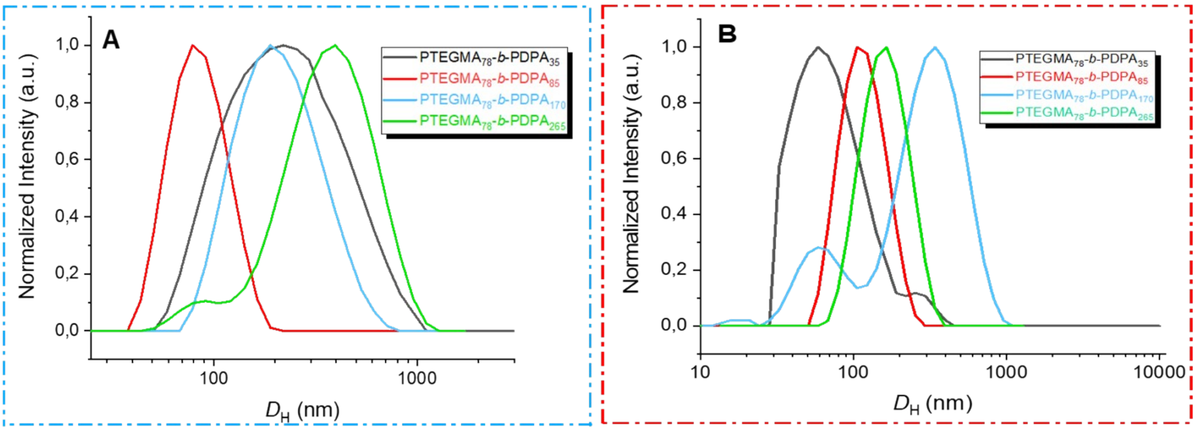

Figure S4. DLS particle size distributions for PTEGMA$_{78}$-*b*-PDPA$_n$ (n = 35, 85, 170 and 265) NPs synthesized at polymerization temperatures (**A**) 45 °C and (**B**) 70 °C.

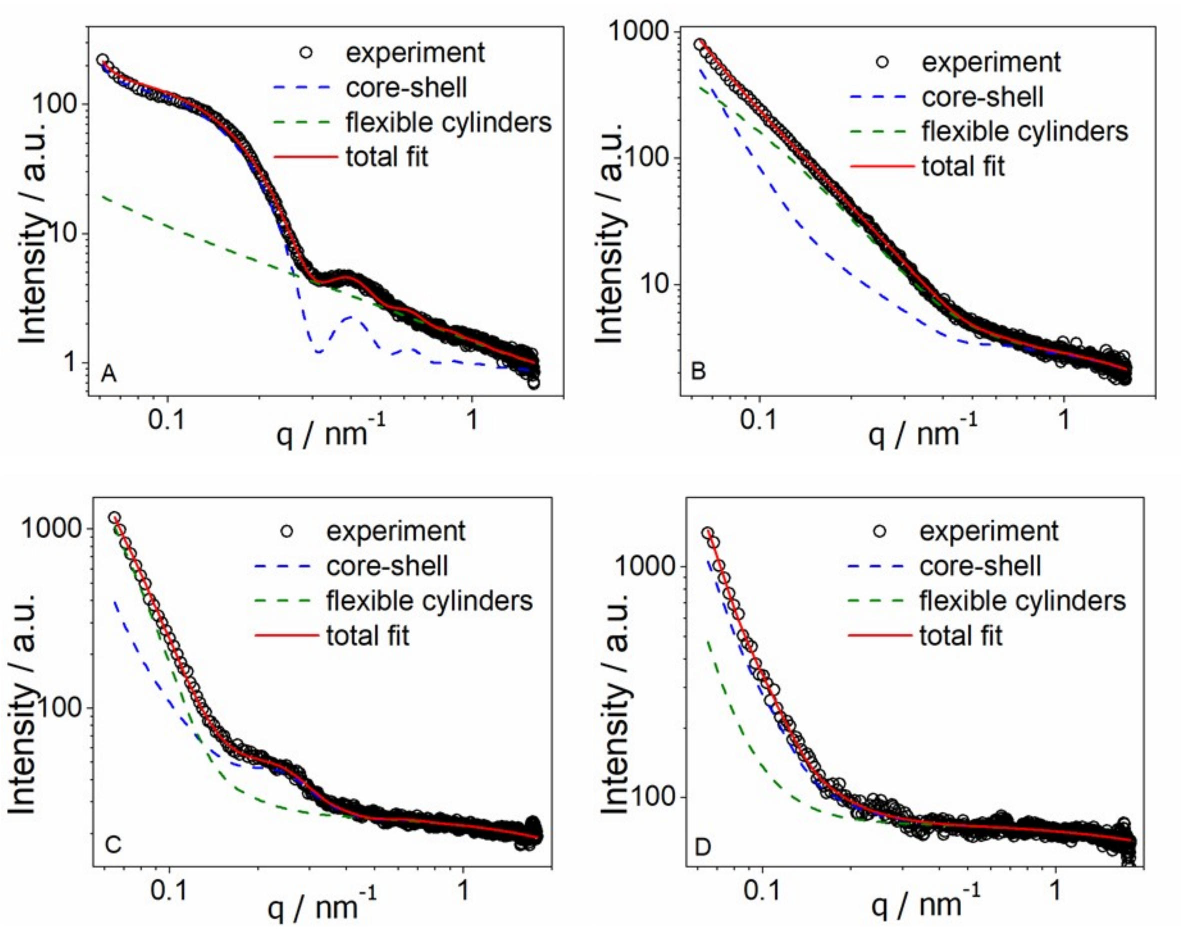

Figure S5. Contribution of each model (core-shell and flexible cylinders) towards the total fit of experimental SAXS data of different PTEGMA$_{78}$-*b*-PDPA$_n$ NPs. (**A**) entry 2, (**B**) entry 3, (**C**) entry 6, (**D**) entry 7, in Table 3.
